# Nanomotor‐Derived Porous Biomedical Particles from Droplet Microfluidics

**DOI:** 10.1002/advs.202104272

**Published:** 2021-11-23

**Authors:** Yuxiao Liu, Yi Cheng, Cheng Zhao, Huan Wang, Yuanjin Zhao

**Affiliations:** ^1^ Department of Rheumatology and Immunology Institute of Translational Medicine The Affiliated Drum Tower Hospital of Nanjing University Medical School Nanjing 210008 China; ^2^ State Key Laboratory of Bioelectronics School of Biological Science and Medical Engineering Southeast University Nanjing 210096 China; ^3^ Department of Vascular Surgery The Affiliated Drum Tower Hospital of Nanjing University Medical School Nanjing 210008 China

**Keywords:** cell culture, droplet, microfluidics, nanomotors, porous microparticles, wound healing

## Abstract

Porous particles have found widespread applications in therapeutic diagnosis, drug delivery, and tissue engineering due to their typical properties of large surface area, extensive loading capacity, and hierarchical microstructures. Attempts in this aspect are focusing on the development of effective methods to generate functional porous particles. Herein, a simple droplet microfluidics for continuously and directly generating porous particles by introducing bubble‐propelled nanomotors into the system is presented. As the nanomotors can continuously generate gas bubbles in the unsolidified droplet templates, the desirable porous microparticles can be obtained after droplet polymerization. It is demonstrated that the generation process is highly controlled and the resultant microparticles show excellent porosity and monodispersity. In addition, the obtained porous microparticles can serve as microcarriers for 3D cell culture, because of their characteristic porous structures and favorable biocompatibility. Moreover, owing to the existence of oxygen in these microparticles, they can be used to improve the healing effects of wounds in the type I diabetes rat models. These remarkable features of the generation strategy and the porous microparticles point to their potential values in various biomedical fields.

## Graphical abstract

The biomedical porous microparticles can be generated through simple droplet microfluidics by introducing bubble‐propelled nanomotors into the system. These particles can serve as microcarriers for 3D cell culture due to their porous structures and favorable biocompatibility; they also can promote wound healing process in the type I diabetes rat models owing to the existence of oxygen.

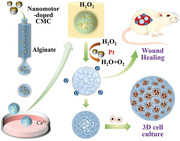

## 1 Introduction

Owing to the characteristic features of enhanced surface area, excellent loading capacity, and hierarchical architecture, porous particles have demonstrated great values in various biomedical fields, such as multiplexed bioassays, controlled drug delivery, noninvasive diagnosis, cosmetic industry, pollutants absorption and removal, protection and encapsulation of active species, tissue engineering, etc.^[^ ^1^, ^2^, ^3^, ^4^, ^5^, ^6^, ^7^ ^]^ The most commonly used approach for generating porous particles is template‐guided fabrication, which is usually implemented by employing desired materials to negatively replicate the original templates.^[^ ^8^, ^9^ ^]^ During this procedure, the appropriate calcination or chemical etching is usually inevitable so as to selectively remove the templates, which is time‐consuming and may impair the structure construction and the encapsulated substances because of the residual toxic etchants.^[^ ^10^, ^11^ ^]^ As an alternative, emulsion‐based technique is a popular method for porous particle generation, specifically including phase inversion, phase ripening, and phase separation.^[^ ^8^, ^12^ ^]^ Compared with the template method, this technique does not require removing process, and thus there is no extra etchants or pore‐forming agents needed, which indicates the emulsion‐based procedure is biologically safer to some extent.^[^ ^13^, ^14^ ^]^ However, the generation devices used in this method always need elaborate design that is not easy‐operating, and the diffusion of organic solvents during the process can also affect the biocompatibility of the generated particles, which might hamper their further applications in biomedicine.^[^ ^15^, ^16^ ^]^ Therefore, new approaches with features of high efficiency, operability, and biosafety, for generating porous particles are still urgently anticipated.

**Figure 1:**
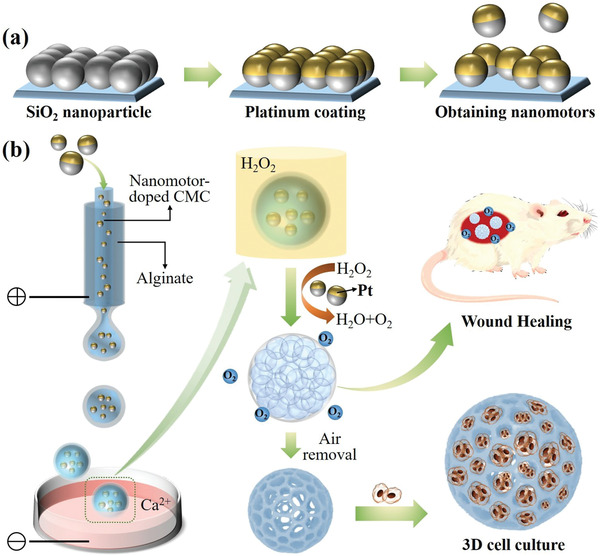
Schematic illustrations: a) the generation process of PSNs; b) the generation process of the porous microparticles and their applications.

In this paper, we present a simple droplet microfluidics for continuously and directly generating porous particles by introducing nanomotors into the system, as schemed in **Figure** **1**. Natural bio‐nanomotors have existed for over millions of years during the evolutionary process, which have exhibited significant capacities in precisely performing complex tasks and establishing the foundation of ecosystem.^[^ ^17^, ^18^, ^19^ ^]^ The attempts of developing artificial nanomotors focus on the design of micro‐ or nanoparticles, i.e., the devices that can convert different energies into movement or force.^[^ ^18^, ^20^, ^21^, ^22^ ^]^ Many researches have been carried out in this field, and these artificial motors have found great potentials in therapeutic diagnosis, target drug delivery, environmental cleanup, etc.^[^ ^23^, ^24^, ^25^, ^26^, ^27^ ^]^ Specifically, the motion of these micro‐ or nanomotors can be either from chemical self‐propulsion or eternal energy propulsion.^[^ ^18^ ^]^ Among the self‐propelled mechanisms, the bubble propulsion is a typical form, in which the movement of motors is propelled by the gas bubbles generated though catalytic or noncatalytic reactions.^[^ ^28^, ^29^, ^30^ ^]^ Therefore, it is conceivable that if the bubble‐propelled nanomotors are introduced into the microfluidic droplet generating system, the gas bubbles will be simultaneously generated in the unsolidified droplets, and thus porous microparticles are able to be obtained after appropriate polymerization means.

For this purpose, platinum (Pt)‐coated silica nanoparticles (PSNs) were used as the specialized nanomotors and the gas bubble generators, because the PSNs could continuously generate oxygen bubbles when they were exposed in the hydrogen peroxide (H_2_O_2_) solution. In this process, a microfluidic electrospray device was implemented to form the core–shell droplets, where the PSNs were dispersed in carboxymethylcellulose sodium (CMC‐Na) solution to form the core compartment while the sodium alginate (Na‐Alg) solution was introduced to form the outer layer. When the CMC‐Na core met with H_2_O_2_ solution, the bubble‐containing droplets could be generated, which were finally polymerized into the desired porous microparticles. It was demonstrated that the generation process was well controlled and the generated porous microparticles showed highly uniform morphologies because of the advantages of microfluidic technology.^[^ ^16^, ^31^, ^32^, ^33^, ^34^ ^]^ Due to the characteristic porous microstructure and the favorable biocompatibility of the resultant microparticles, they were ideal for serving as the microcarriers of 3D cell culture, which even could be beneficial to the further applications like tissue engineering. Attractively, owing to the oxygen bubbles arising during the generation process and the pivotal role of oxygen in promoting cell proliferation and tissue reconstruction,^[^ ^35^, ^36^, ^37^, ^38^ ^]^ the obtained oxygen‐containing porous microparticles could also be used for accelerating wound healing. These results indicate that our strategy for generating porous microparticles is feasible and efficient, and the resultant microparticles also have promising prospects in biomedical fields.

## 2 Results and Discussion

**Figure 2:**
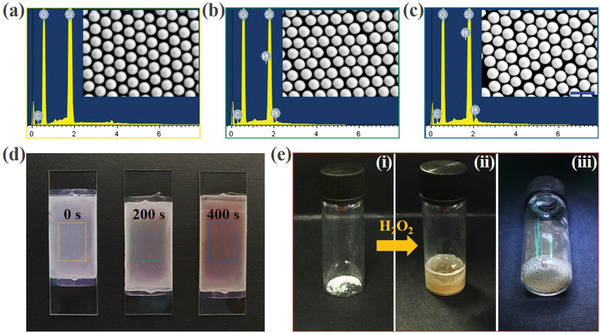
Fabrication of the bubble‐propelled nanomotors: a) SEM images (inset) and the corresponding elemental analysis of the unmodified SN monolayer, b) the SN monolayer after Pt sputtering for 200 s, and c) the SN monolayer after Pt sputtering for 400 s; d) the structural color of the SN monolayers after Pt sputtering for 0, 200, and 400s, from left to right; e) performance of the PSNs after meeting with the H_2_O_2_ solution. The scale bar is 500 nm.

In a typical experiment, the bubble‐propelled nanomotors were first fabricated. To accomplish the fabrication process, two steps were included in the strategy. First, the self‐assembled monolayers of silica nanoparticles (SNs) were achieved by depositing these particles on a cleaned glass slide. The microstructure of the monolayers was demonstrated in **Figure** **2**a, and an obvious ordered hexagonal packing structure was observed in this film, which was formed by the self‐assembly of the SNs during the deposition process. The next step was depositing Pt layer on the SNs, and it was achieved by introducing the glass slide with SN monolayer into a vacuum chamber where the nanoparticles could be coated by Pt atoms through a sputterer. To verify whether the Pt atoms were successfully deposited on the SNs, the scanning electron microscope (SEM) images of the monolayers after sputtering were also obtained (Figure 2b,c). It was found that the nanoparticles coated by Pt layer demonstrated brighter view under the SEM observation compared with the image of unmodified SNs, which could be ascribed to the enhancing electric conductivity after metal sputtering. In addition, the corresponding elemental analysis also verified that the Pt layer was effectively deposited on the SNs (Figure 2a–c). Besides, because the Pt element has a dark color, the Pt‐coated SN monolayers demonstrated higher color saturation. Specifically, compared with the uncoated monolayer, the thicker the Pt layer was, the higher color saturation the coated SN monolayer would have, as shown in Figure 2d. Finally, the designed PSNs were removed from the glass slide through ultrasound treatments to obtain the discrete nanomotors. We tested the performance of the designed PSNs when they were exposed to the H_2_O_2_ solution, and it was demonstrated that a large number of gas bubbles could be continuously generated (Figure 2e), which showed these particles were capable of serving as bubble‐propelled nanomotors for the following experiments.

**Figure 3:**
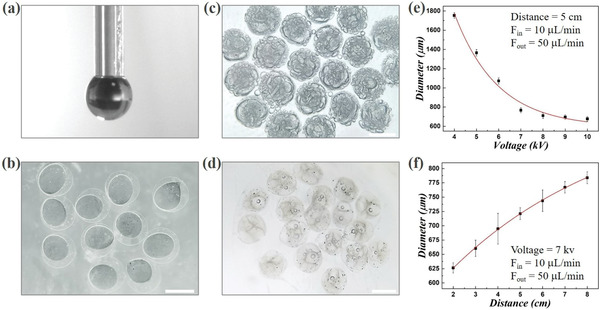
Generation of the core–shell microcapsules and porous microparticles: a) the real‐time image of the orifice of the microfluidic electrospray device during the microcapsule generation process; b) the microcapsules with a solidified shell layer and an unsolidified inner core; c) the porous microparticles containing extensive oxygen bubbles; d) the porous microparticles without or with few bubbles; e,f) the relationships of the microcapsules’ diameter to the voltage of the electric field and the collection distance, respectively. The scale bars are 500 µm.

To generate the oxygen bubble‐containing porous microparticles, the core–shell microcapsules were first prepared by using the microfluidic electrospray system. The specific microfluidic device was fabricated through assembling two cylindrical capillaries coaxially, where two different (inner and outer) phases were introduced to generate a sequential co‐flow regime, respectively (**Figure** **3**a). Specifically, the inner and outer phases were PSNs‐dispersed CMC‐Na solution and Na‐Alg solution, respectively. As shown in Figure 3a, when these different liquids were forced into the microfluidic device through corresponding microchannels, the outer phase would be sheathed around the inner phase at the orifice of the capillaries due to the hydrodynamic focusing. Then the flows were broken up into discrete double‐emulsion droplets under an external applied electric field, and the Na‐Alg solution in the shell layer would be first gelled with calcium chloride (CaCl_2_) solution in the collection container to maintain the microcapsules’ morphology, as shown in Figure 3b and Figure S1 (Supporting Information). Due to the advantages of microfluidic electrospray method, the generation process of the microcapsules was highly controlled, and the overall sizes of these capsules could also be modulated by simply adjusting the voltage of the electric field, the collection distance, and the flow rates of accordant fluid phases (Figure 3 and Figure S1, Supporting Information). The experimental results showed that the size of the generated microcapsules would obviously decrease with the increasing voltage of the external applied electric field, while it would increase when the collection distance gradually grew. In addition, the flow rates of the inner and outer phases could also affect the diameter of the capsules, and it was found that their size was directly proportional to the flow rates. Besides, the monodispersity of the resultant microcapsules was also investigated. It was found that these capsules demonstrated uniform sizes and morphologies, and the coefficient of variation (CV) of the size distribution of these capsules was below 5%, which indicated that the resultant microcapsules were highly monodispersed, as shown in Figure S2 (Supporting Information).

**Figure 4:**
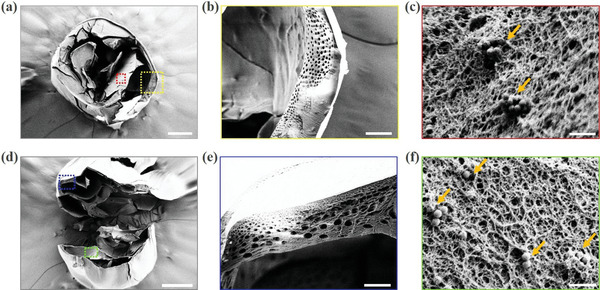
Microstructures of the porous microparticles: a) the entire view of the porous microparticle under SEM observation; b) the enlarged view of the yellow frame in (a); c) the enlarged view of the red frame in (a) and the PSNs remained in the porous microparticles (labeled by the orange arrowheads); d) the interior microstructure of the resultant porous microparticle; e) the microstructure of the framework of the pore structure (enlarged view of the blue frame in (d)); f) the enlarged view of the green frame in (d) the PSNs remained in the porous microparticles (labeled by the orange arrowheads); the scale bars are 200 µm in (a, d), 100 µm in (b), 1 µm in (c, f), and 50 µm in (e).

Afterward, the porous microparticles were produced based on the microcapsules fabricated in the previous step. As described above, the shell layer of the microcapsules would be first solidified through the gelation reaction of Na‐Alg and CaCl_2_ solution. At that time, the CMC‐Na solution in the core compartment was not solidified yet, and thus when the obtained microcapsules were exposed in the environment full of H_2_O_2_, extensive oxygen bubbles would be produced in the capsules after the H_2_O_2_ solution diffused into the inner core, as shown in Figure 3c. In addition, Figure S3a–c (Supporting Information) showed the gradual formation and increase of oxygen bubbles in the microcapsules in detail, demonstrating how the microcapsules finally became the oxygen‐containing porous microparticles. Also, it was found that after vacuum treatment or standing for a period, the oxygen bubbles in the microparticles would gradually be discharged, thereby forming porous particles without containing gas, as demonstrated in Figure 3d and Figure S3d–f (Supporting Information). The more detailed microstructures of the resultant porous microparticles were also observed by the SEM, as demonstrated in **Figure** **4**. It was confirmed that the designed microparticles had a typical external‐to‐internal connected porous structure with many windows on the surface of the particles, as shown in the entire view of the resultant particle in Figure 4a–d. Moreover, in the interior of these particles, there were many smaller holes existing among the larger pores, which indicated that the generated pores were also interconnected with each other (Figure 4b,e and Figure S4, Supporting Information). This characteristic could benefit these particles serving as microcarriers for 3D cell culture in the future, since these interconnected openings could contribute to the substances exchange and nutrient transport. Besides, through the SEM characterization, the PSNs could also be observed in the microparticles (Figure 4c,f), indicating that after completing the preparation of the porous microparticles, a few nanomotors would remain in the framework of the pore structure.

**Figure 5:**
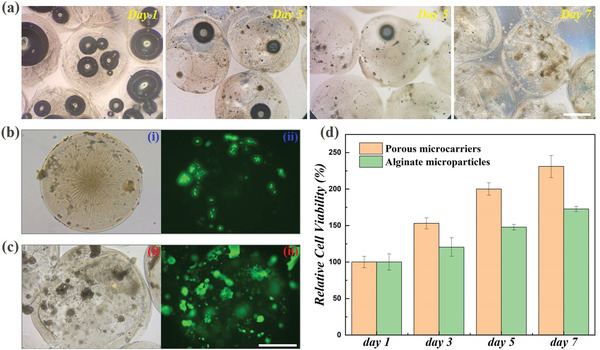
Porous microparticles for 3D cell culture: a) the conditions of the cells in the porous microparticles after culturing for 1, 3, 5, and 7 d; transmission optical microscope images (i) and fluorescent images (ii) of the b) solid alginate‐CMC microparticles and c) porous microparticles after cocultured with 3T3 cells for 7 d; d) the results of the MTT assay for solid alginate‐CMC microparticles and the porous microparticles cocultured with 3T3 cells. The scale bars are 500 µm.

To verify the capacity of the prepared porous microparticles for 3D cell culture, the resultant microparticles were then used as microcarriers for culturing the standard fibroblast cell line (3T3) cells. It was demonstrated that the 3T3 cells could enter the interior of the microparticles through the windows of the pores, and as the oxygen bubbles were gradually expelled from the particles, the cells also gradually aggregated inside the pores. The whole formation process of the cell aggregates was observed and demonstrated in **Figure** **5** and Figure S5 (Supporting Information). Owing to the transparent property of alginate and CMC hydrogel, it could be easily observed that after 7 d culture, there were many cell aggregates in the pores of the microparticles. Moreover, we also compared the cell growing conditions between the designed porous microparticles and the solid alginate microparticles. The transmission optical images and the fluorescent images all showed that the 3T3 cells could form aggregates and proliferated much better in the porous microparticles, while there were merely few cells that could adhere and grow on the surface of the solid microparticles owing to the relatively poor adhesivity of the alginate hydrogel, as shown in Figure 5b,c. The MTT assays were also carried out to quantitatively compare the cell viability and cell proliferation in these two different kinds of microparticles (Figure 5d). The results showed that the 3T3 cells cultured in the porous microcarriers exhibited much better viability and proliferation rate than cells in the solid alginate microparticles, which indicated the advantages of the resultant porous microparticles for facilitating cell growth. Thus, our designed porous microcarriers were suitable for 3D cell culture and cell spheroids formation, which had great potentials in wound healing, cell therapy, and tissue regeneration.

**Figure 6:**
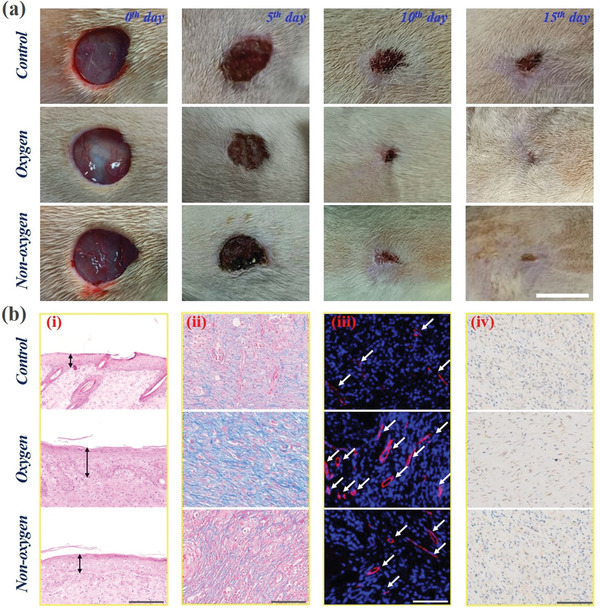
Investigation of the capacity of the oxygen‐containing porous microparticles in promoting wound healing: a) the changes of the wound areas in the control, oxygen and non‐oxygen groups during the wound healing process. The scale bar is 1 cm; b) histological analysis: (i) the results of H&E staining analysis; (ii) the results of Masson staining analysis; (iii) the results of double immunofluorescent staining of CD31 plus *α*‐smooth muscle actin (*α*‐SMA); (iv) the results of immunohistochemistry staining of IL‐6. The scale bars are 200 µm in (i) and 100 µm in (ii, iii, iv).

To analyze the practical value of the designed oxygen‐containing porous microparticles for promoting wound healing effects, we investigated their performance in repairing the full‐thickness cutaneous wounds in type I diabetes rat models. The animal models used in this experiment were established by creating a circular wound with a diameter of 1 cm on the back of the experimental rat. These animal models were then randomly divided into three groups, i.e., one group that received no extra treatment (Control group), one group that was treated by the oxygen‐containing porous microparticles (oxygen group), and one group that was treated by the solid microparticles derived from the alginate‐CMC composite hydrogel (non‐oxygen group), as shown in Figure S6 (Supporting Information). Later, the whole wound healing process was recorded, and the corresponding changes of wound areas were demonstrated in **Figure** **6**a. It was found that the wounds in the oxygen group were almost completely healed on the 15th d, demonstrating a much better repairing condition compared with the other two groups. This phenomenon could be ascribed to that the designed porous microparticles were able to provide sufficient oxygen to the wounds, and a key factor for the difficulties in healing the wounds of diabetes is the lack of oxygen, and thus such an oxygen‐containing microparticles could effectively promote wound healing. Besides, the composite hydrogel could provide sufficient hydration that allowed the wounds to remain moist and effectively enhanced the tissue regeneration. This feature, together with its favorable biocompatibility, made the healing condition of the wounds in the non‐oxygen group was slightly better than that in the control group, which confirmed the importance of hydrogel in the wound healing process. Moreover, the changes of the wound areas were also quantitatively measured, as shown in Figure S7a,b (Supporting Information). The results showed that the constriction extent of the wounds in the oxygen group was much higher than that in the control group and the non‐oxygen group, while the ratio of the wound site in the non‐oxygen group was relatively smaller than that in the control group, which was consistent with the previous discussion.

In addition to directly observing the changes of wound areas, the hematoxylin and eosin (H&E) staining analysis was also implemented to characterizing the development of epithelization, as demonstrated in Figure 6b (i). Specifically, the observation of the regenerated epithelial thickness could also indicate the wound recovery conditions. Generally, a thicker regenerated epithelial tissue represents the better healing of the wounds, and the H&E staining images demonstrated that the regenerated epithelial tissue was thickest in the oxygen group, followed by the non‐oxygen group and the control group successively. Figure S7c (Supporting Information) demonstrated the corresponding statistical analysis of the regenerated epithelial thickness in different groups, further corroborating the conclusions directly obtained from observing the H&E staining images. Moreover, the Masson staining, double immunofluorescent staining of CD31 plus *α*‐smooth muscle actin (*α*‐SMA), and immunohistochemistry staining of IL‐6 were also conducted to further investigate the conditions of collagen deposition, angiogenesis, and inflammation in different groups after the wound healing process. As shown in Figure 6b (ii), the results of Masson staining showed that the collagens demonstrated extensive deposition and a highly directional alignment in the oxygen group, which proved the promoted extracellular matrix reconstruction and the tissue remodeling. Besides, the immunofluorescent staining analysis displayed that the highest blood vessel density was observed in the wound bed in the oxygen group (Figure 6b (iii) and Figure S7d, Supporting Information), which proved from the side that this group had the best tissue restoration condition, because the angiogenesis could allow adequate substances supply and was essential in tissue regeneration. Additionally, the immunohistochemistry staining analysis also demonstrated that merely a small quantity of IL‐6 secretion was observed in the oxygen group, indicating that there were just slight inflammation or infection in this group, as shown in Figure 6b (iv). These features pointed to the desirable ability of the designed oxygen‐containing porous microparticles in promoting wound healing effects.

## 3 Conclusion

In conclusion, we demonstrated a microfluidic electrospray strategy for directly generating porous microparticles through introducing bubble‐propelled nanomotors into the system. The generation process and the particle morphology were highly controllable due to the characteristics of microfluidic technology. More attractively, the resultant porous microparticles could serve as microcarriers for 3D cell culture, where cells exhibited more realistic morphologies and could form aggregates in the pores. Furthermore, the oxygen‐containing porous microparticles could also be used for promoting the wound healing effects. Compared with treatment‐free and non‐oxygen contained solid particles treatment, the specially designed microparticles could effectively improve the healing effects of wounds in the type I diabetes rat models. These unique features indicate the promising prospects of the designed porous microparticles in various biomedical fields, such as substances encapsulation and delivery, novel therapeutics, wound healing, tissue engineering, and organ‐on‐a‐chip.

## 4 Experimental Section

### Materials

The SNs were purchased from NanJing DongJian Biological Technology Co., Ltd. *n*‐Butanol, ethanol, and dimethyl sulfoxide (DMSO) were purchased from Sinopharm Chemical Reagent Co., Ltd. Na‐Alg, CMC‐Na, CaCl_2_, AlCl_3_, and polyvinyl alcohol (PVA) were purchased from Aladdin. H_2_O_2_ solution (3%) was purchased from ANNJET. The 3T3 cells were obtained from the Cell Bank of the Chinese Academy of Sciences, Shanghai, China. Calcein‐AM was purchased from Molecular Probes Co. The MTT reagent was purchased from J&K Scientific Ltd., Shanghai. Streptozotocin (STZ) was purchased from Sigma Aldrich Co. Deionized water with a resistivity of 18.2 M cm^−1^ was obtained from a Millipore Milli‐Q system.

### Fabrication of the Nanomotors

In this section, the monolayer of SNs was first produced. The SNs were dispersed in *n*‐butanol, and the dispersion was purified through multiple centrifugations. The SN dispersion with a concentration of 20 wt% was then mixed with ethanol at a volume ratio of 2:1. A clean glass slide was diagonally placed at the bottom of the customized container, and the water was subsequently added into the container until totally covering the glass slide. Then the mixture of SN dispersion and ethanol was dripped onto the water surface, on which the SNs could be self‐assembled into an ordered 2D array. Later, open the valve at the bottom of the container to gradually drain the water, and the SNs assembled array could deposit on the glass slide. After drying at the room temperature, the ordered monolayer of SNs could be finally obtained. Next, the glass slide with SN monolayer was platinized through a sputter coater. Finally, the discrete PSNs, i.e., the nanomotors, were obtained by removing the nanoparticles from the glass slide through scraping and ultrasound treatment.

### Generation of the Core–Shell Microcapsules

To generate the core–shell microcapsules, the microfluidic electrospray system was used. In this section, the microfluidic chip was first fabricated. Two cylindrical capillaries were coaxially assembled, in which the inner diameter of the outer and inner capillary was 800 and 300 µm, respectively. The orifices of the outer capillary and inner capillary were basically flush. The connection points of the microfluidic chip were sealed by syringe needles and epoxy resin. The outer phase fluid (1% Na‐Alg solution) and the inner phase fluid (1% CMC‐Na solution containing 2% PVA and dispersed with PSNs) were introduced into the microfluidic chip through syringe pumps. A voltage power supply was connected between the syringe needle on the chip and the collecting pool. The collecting pool was full of CaCl_2_ solution (2%). Through adjusting the electric voltage, collection distance, and flow rates, the size of the generating microcapsules could be modulated.

### Generation of the Porous Microparticles

After the core–shell microcapsules were generated, the obtained capsules were put into the H_2_O_2_ solution (3%). When the H_2_O_2_ solution was diffused into the inner core, the oxygen bubbles would gradually produce and finally fill the entire microcapsules. After the capsules were full of oxygen bubbles, they were then put into the AlCl_3_ solution (4%) to solidify the CMC hydrogel and prevent the fusion of bubbles through the ionotropic gelation process, and this process would immediately occur when CMC‐Na met the crosslinking agent (AlCl_3_ solution). Through the vacuum treatment or a period of standing, the bubbles could be gradually expelled from the generated oxygen‐containing porous microparticles. The resultant porous microparticles were washed by deionized water and then freeze‐dried through a freeze dryer, and the obtained samples were characterized by the SEM (Hitachi S3000 N).

### Porous Microparticles for 3D Cell Culture

In this section, the performance of the porous microparticles and the solid alginate‐CMC microparticles in 3D cell culture was compared. To obtain the alginate‐CMC microparticles, the 1% Na‐Alg solution and 1% CMC‐Na solution (containing 2% PVA and dispersed with PSNs) were mixed, and the mixture was introduced into a simple microfluidic electrospray device to form into the single‐emulsion droplet, and finally solidified into solid microparticles through the gelation of AlCl_3_ in the collection pool. To investigate the performance of these microparticles, the porous and solid particles were thoroughly washed by deionized water and PBS buffer and were placed into the ultraclean desk and irradiated by the UV light for 4 h before the cell experiments. After UV disinfection, these microparticles were put into the 48‐well plate (eight particles in each hole). Then, the 3T3 cells were homogeneously dispersed in the culture media and cocultured with the microparticles. After culturing for 7 d, the cells were stained by Calcein‐AM and were observed through the inverted fluorescence microscope (IX71, Olympus, Japan). The quantitative MTT assay was implemented after cell seeding for 1, 3, 5, and 7 d. The specific steps of MTT assay are as follows: the cell‐cultured microparticles were first transferred into another clean hole and then the culture medium was added with 10% MTT solution into the hole. After incubation at 37 °C for 4 h in the cell incubator, the culture medium was removed, and 400 µL DMSO was subsequently added into each hole to dissolve the formazan crystals. After complete dissolution, the solution was transferred into a 96‐well plate (100 µL in each well), and the plate was read by a microplate reader (SYNERGY|HTX).

### Porous Microparticles for Wound Healing

In this section, the animal model should be first established. All animal experiments were performed in strict accordance with the Guide for the Care and Use of Laboratory Animals and have received approval from The Affiliated Drum Tower Hospital of Nanjing University Medical School. The SD rats (male, weighing about 200 g, obtained from Sino‐British SIPPR/BK Lab. Animal Ltd.) were first adaptive fed for one week. After fasting overnight, the STZ (1% w/v, dissolved in citrate buffer, pH = 4.2–4.5) was intraperitoneally injected into the rats (70 mg kg^−1^) to induce the type I diabetes in the SD rats. If monitoring the fasting blood glucose of the rats to reach more than 17 mmol L^−1^ for one week, it is indicated that the model is successfully established. After the rats were anesthetized with isoflurane, their backs were shaved, and a circular full‐thickness cutaneous wound with a diameter of 1 cm was created by scissors. Then the rat models were randomly divided into three groups. The first group was control group, in which the rats did not receive any treatments; the second group was the oxygen group, in which the rats were treated by the oxygen‐containing porous microparticles; the third group was the non‐oxygen group, in which the rats were treated by the solid alginate‐CMC microparticles. Microparticles were laid flat on the wound surface and replaced daily to ensure adequate oxygen supply. On the 0th, 5th, 10th, and 15th d, the photographs of the wound shape were taken to record the recovery condition of the wound area, and the final wound healing area was analyzed by using ImageJ Software. After 15 d, all rats were sacrificed, the wound bed and the surrounding tissue were taken out and fixed by 4% paraformaldehyde. Then the tissue samples were dehydrated, embedded in paraffin, and made into sections. The H&E staining, Masson staining, immunohistochemical staining, and immunofluorescence staining were implemented to carry out the corresponding analysis.

## Conflict of Interest

The authors declare no conflict of interest.

## Supporting information

Supporting Information

## Data Availability

Research data are not shared.
