# Supplementary material for: Nanomotor‐Derived Porous Biomedical Particles from Droplet Microfluidics
Source: Adv Sci (Weinh). 2021 Nov 23;9(4):2104272. doi: 10.1002/advs.202104272 (PMC8811803; doi:10.1002/advs.202104272)
Supplement: Supplementary file 1 — Supporting Information [file ADVS-9-2104272-s001.pdf]

WILEY-VCH

ADVANCED SCIENCE
Open Access

Supporting Information

for *Adv. Sci.*, DOI: 10.1002/advs.202104272

Nanomotor-derived porous biomedical particles from droplet microfluidics

*Yuxiao Liu, Yi Cheng, Cheng Zhao, Huan Wang, Yuanjin Zhao**

Copyright WILEY-VCH Verlag GmbH & Co. KGaA, 69469 Weinheim, Germany, 2018.

Supporting Information

Nanomotor-derived porous biomedical particles from droplet microfluidics

*Yuxiao Liu, Yi Cheng, Cheng Zhao, Huan Wang, Yuanjin Zhao**

Dr. Y. X. Liu, Dr. Y. Cheng, Dr. C. Zhao, Prof. Y.J. Zhao
Department of Rheumatology and Immunology, Institute of Translational Medicine, The Affiliated Drum Tower Hospital of Nanjing University Medical School, Nanjing 210008, China
Email: yjzhao@seu.edu.cn

Dr. Y. X. Liu, Dr. C. Zhao, Dr. H. Wang, Prof. Y.J. Zhao
State Key Laboratory of Bioelectronics, School of Biological Science and Medical Engineering, Southeast University, Nanjing 210096, China

Dr. Y. Cheng
Department of Vascular Surgery, The Affiliated Drum Tower Hospital of Nanjing University Medical School, Nanjing 210008, China

Author contributions:

Y.J.Z. conceived the idea and modified the paper. Y.X.L. and Y.C. carried out the experiments and analyzed data. Y.X.L. wrote the paper. C.Z. and H.W. assisted with data analysis and paper writing. The authors declare no competing financial interests.

Supporting Figures:

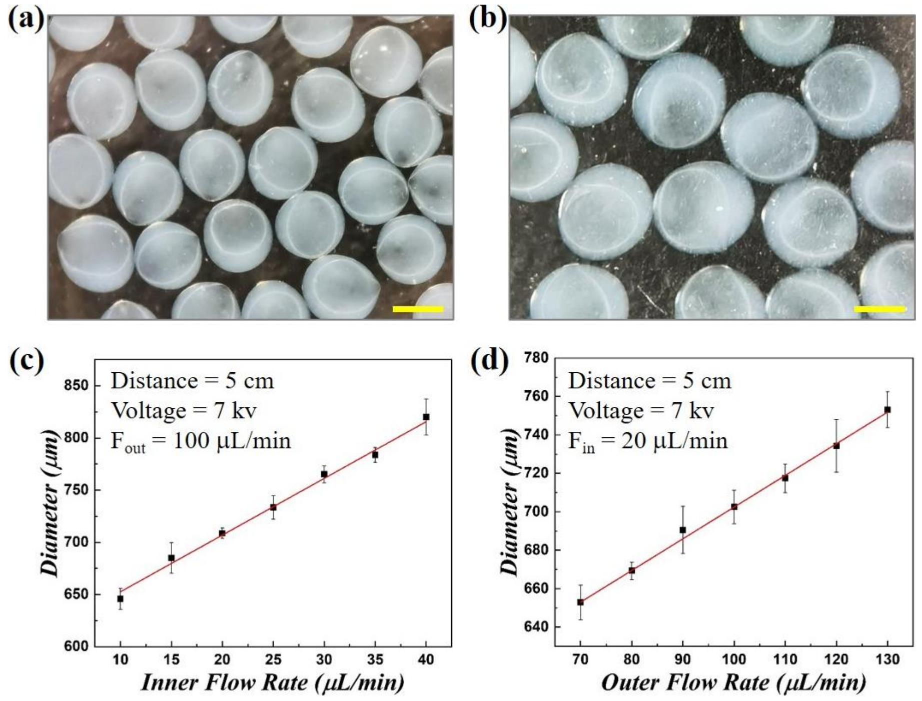

Figure S1. (a-b) The microscope images of the core-shell microcapsules with different diameters; (c-d) the relationship between the microcapsules' diameter and the inner (c)/outer (d) flow rates. The scale bars are 500 μm.

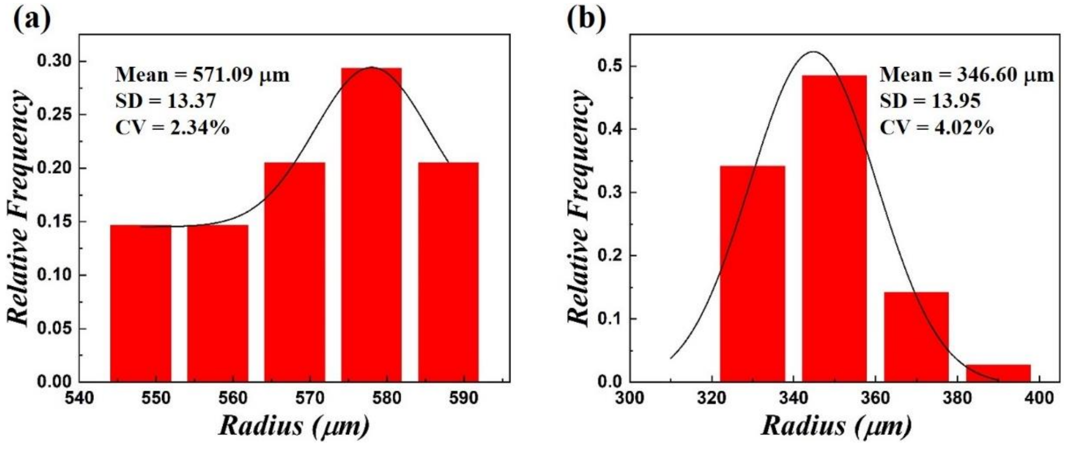

Figure S2. The statistical size distributions of the microcapsules derived from different voltages of the external applied electric field: (a) 5 kV; (b) 10 kV.

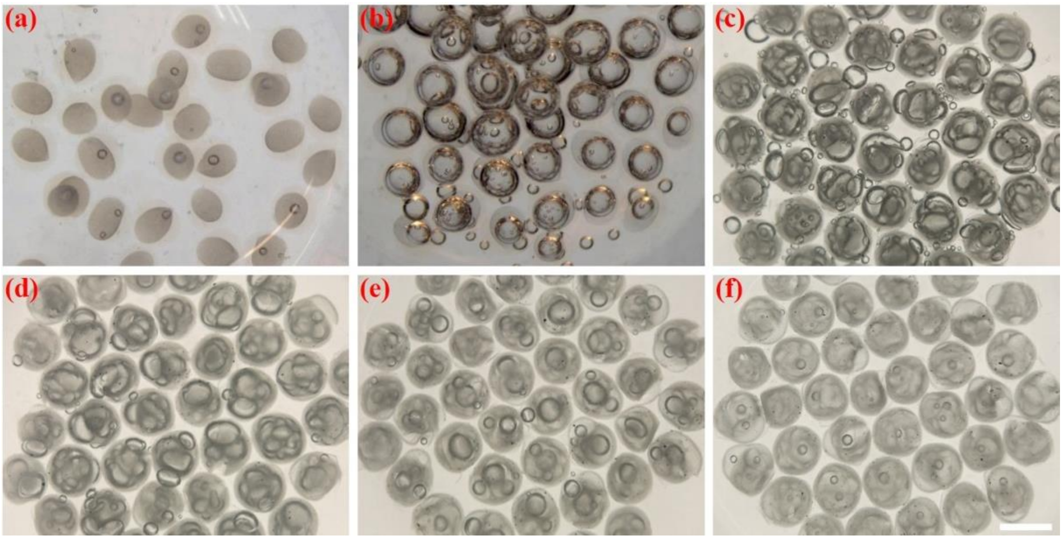

Figure S3. (a-c) the oxygen bubbles gradually increased in the microcapsules; (d-f) the oxygen bubbles were gradually expelled from the porous microparticles after vacuum treatment. The scale bar is 500 μm.

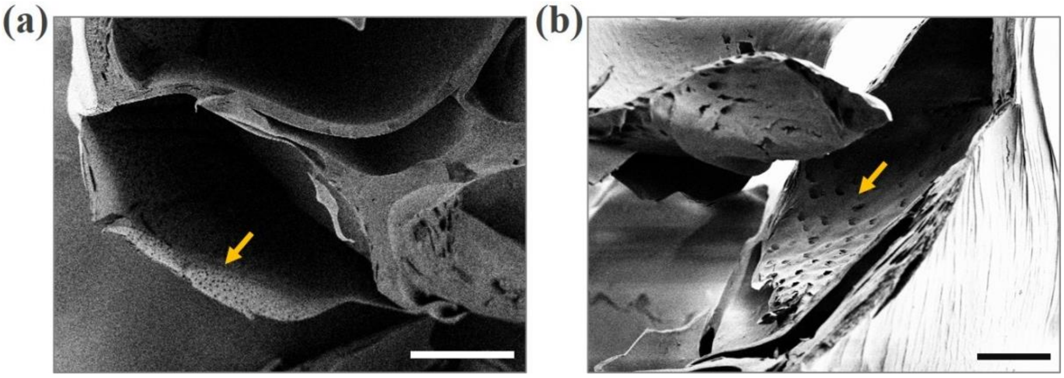

Figure S4. The enlarged view of the smaller holes (labelled by the arrowheads) in the larger pores. The scale bars are 200 μm in (a) and 100 μm in (b).

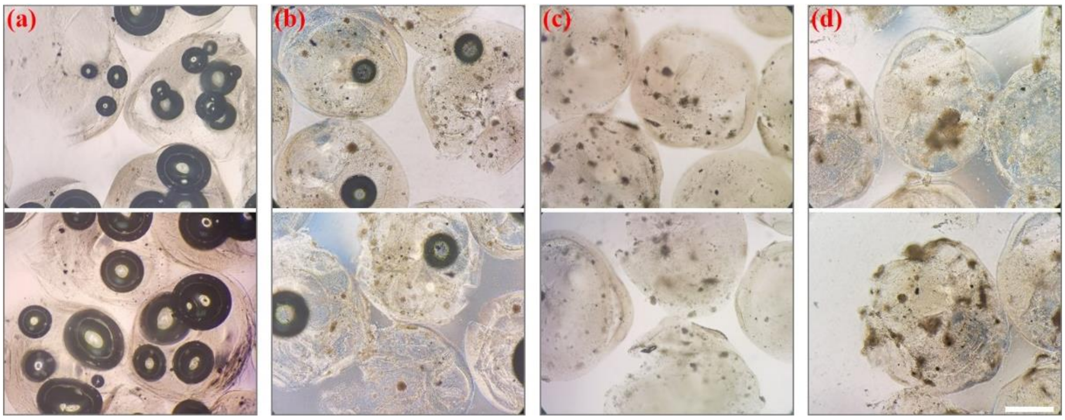

Figure S5. The 3T3 cells conditions in the porous microparticles after culturing for 1 day (a), 3 days (b), 5 days (c), and 7 days (d). The scale bar is 500 μm.

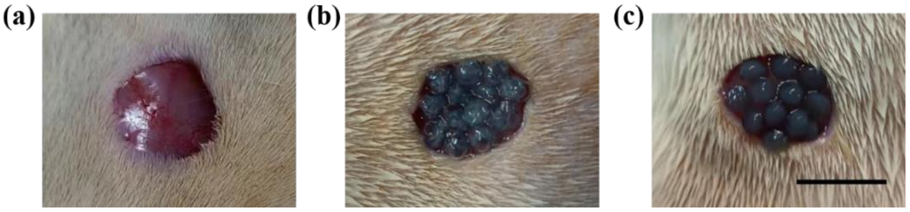

Figure S6. The different treatments of wounds. (a) The control group received no extra treatment. (b-c) The oxygen and non-oxygen groups were treated with oxygen-contained porous microparticles (b) and the solid microparticles (c), respectively. The scale bar is 1 cm.

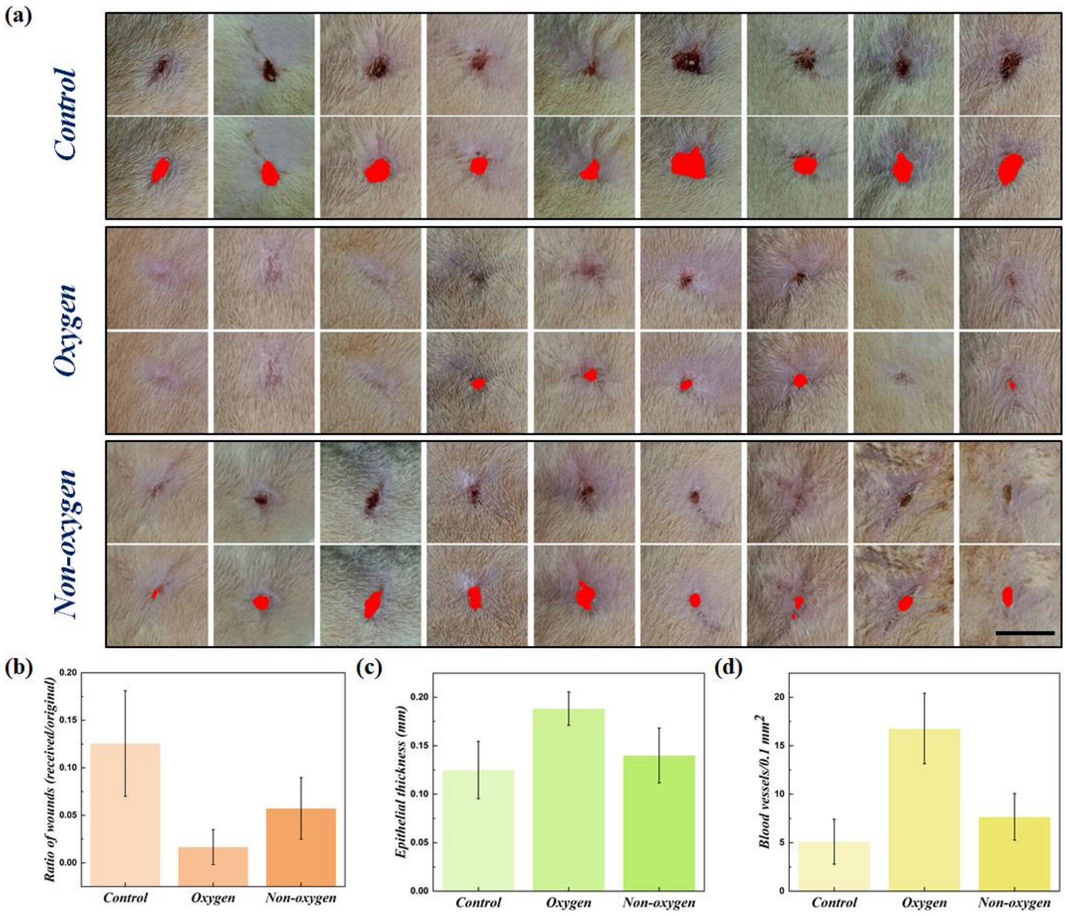

Figure S7. Evaluation of wound healing status. (a) The final wound healing status (upper) and area calculation (bottom). The scale bar is 1 cm. (b-d) The statistical analysis of the ratio of wounds (the wound area of the received sample divided by that of the original modeling), the regenerated epithelial thickness, and the density of the blood vessels after wound healing.
